# Supplementary material for: Microscopic theory, analysis, and interpretation of conductance histograms in molecular junctions
Source: Nat Commun. 2023 Nov 23;14:7646. doi: 10.1038/s41467-023-43169-3 (PMC10667247; doi:10.1038/s41467-023-43169-3)
Supplement: Supplementary file 1 — Supplementary Information [file 41467_2023_43169_MOESM1_ESM.pdf]

Supplementary Information

Microscopic Theory, Analysis, and Interpretation of Conductance Histograms in Molecular Junctions

Leopoldo Mejía[1], Pilar Cossio[2,3,4], and Ignacio Franco[1,5]

[1]Department of Chemistry, University of Rochester, Rochester, NY 14627, USA
[2]Center for Computational Mathematics, Flatiron Institute, New York City, NY 10010, USA
[3]Center for Computational Biology, Flatiron Institute, New York City, NY 10010, USA
[4]Biophysics of Tropical Diseases Max Planck Tandem Group, University of Antioquia, 050010 Medellín, Colombia
[5]Department of Physics, University of Rochester, Rochester, NY 14627, USA

CONTENTS

I. Effects of parameters c_1-c_4 on the conductance histogram S2

II. Effect of the microscopic parameters on the conductance histogram S3

III. Recovery and robustness of the microscopic parameters from fitting S4

IV. Comparison with the Reuter-Ratner model S6

V. Fit to MCBJ experiments S8

Supplementary references S10

I. EFFECTS OF PARAMETERS c_1-c_4 ON THE CONDUCTANCE HISTOGRAM

To better elucidate the role of c_1-c_4 in the conductance histograms, in Fig. S1 we show the effect of independently varying each of the four parameters on the conductance contributions from junction formation and rupture processes (left panels) and the conductance histograms (right panels). Parameters c_1 and c_2 control the junction formation (see Fig. S1a,c) and therefore affect the higher-conductance side of the histograms (Fig. S1b,d) if we assume $\gamma < 0$ (i.e. a conductance that decays with the junction gap). By contrast, parameters c_3 and c_4 control the junction rupture (Fig. S1e,g), and therefore, affect the lower-conductance side of the histograms (Fig. S1f,h) for $\gamma < 0$.

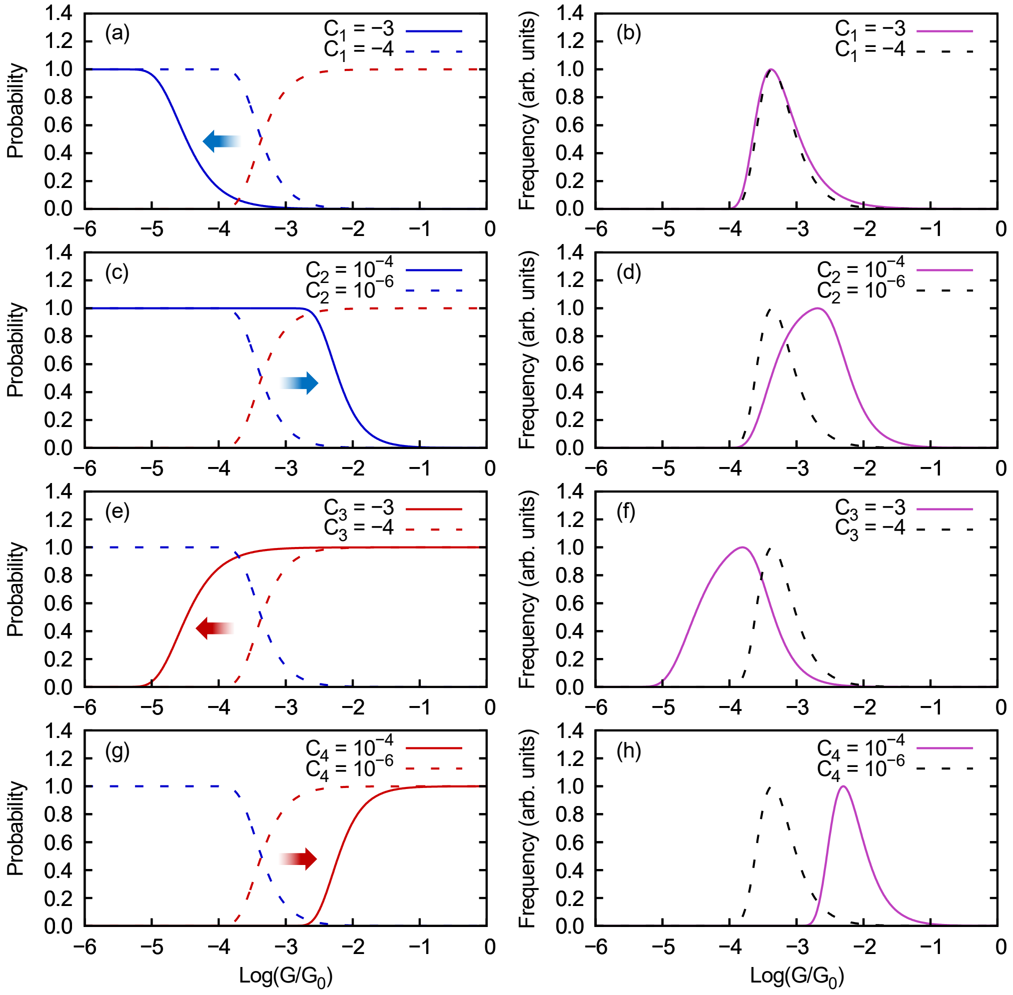

Supplementary Figure S1. Effect of independently varying the fitting parameters c_1-c_4 on the conductance histogram (Eq. 8). The left panels (a, c, e, g) show the conductance probability considering the junction formation only, i.e. $p(\log T)_\mathrm{f} = (1 - \exp[-c_2 \mathrm{e}^{c_1 \log T}])$ (blue lines), and junction rupture only, i.e. $p(\log T)_\mathrm{r} = \exp[-c_4 \mathrm{e}^{c_3 \log T}]$ (red lines). The right panels (b, d, f, h) show the resulting conductance histogram $p(\log T) = p(\log T)_\mathrm{f}\, p(\log T)_\mathrm{r}$, corresponding to Eq. 8. In all cases, we assumed that the conductance decays with the junction gap ($\gamma < 0$). Note that, c_1 and c_2 affect the high-conductance side of the histograms, while parameters c_3 and c_4 affect the low-conductance side. For $\gamma > 0$ the effect is the opposite.

II. EFFECT OF THE MICROSCOPIC PARAMETERS ON THE CONDUCTANCE HISTOGRAM

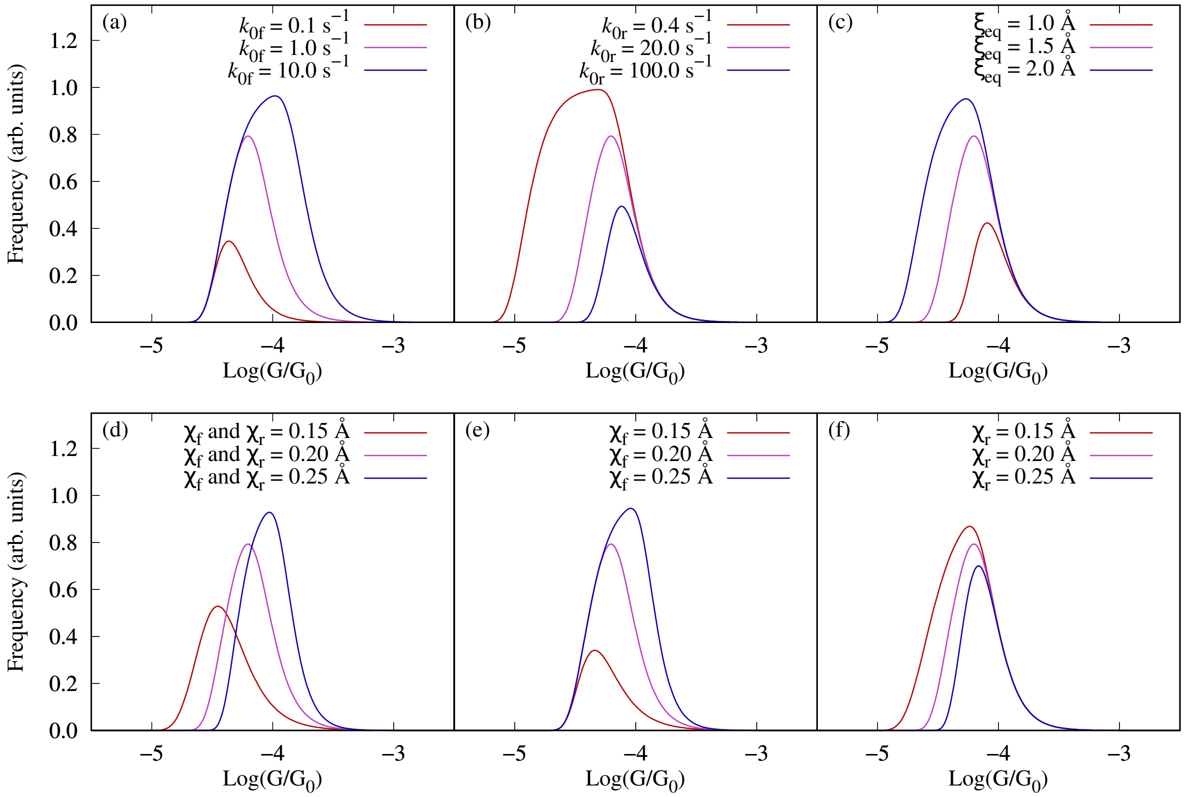

Supplementary Figure S2. Effect of varying the microscopic parameters on the conductance histogram. The panels show the influence of varying (a) metal-metal spontaneous rupture rate; (b) molecular junction spontaneous rupture rate; (c) junction electrode gap at mechanical equilibrium; distance to transition state in the FEP of (d) both the metal-metal contact and molecular junctions, (e) only the metal-metal contact, and (f) only the molecular junction. In all cases, the conductance histogram was calculated using Eq. 8. and the parameters in Table 1 except for the ones being varied.

Figure S2 show the effect of varying the microscopic parameters that define the conductance histogram in our model. This figure complements Fig. 4 in the main text.

Stable metal-metal contacts require the application of higher forces to be ruptured. Thus, metal-metal junctions with smaller spontaneous rupture rates (k_{0f}) lead to longer initial electrode gaps (ξ_0). Therefore, as shown in Fig. S2a, the conductance histogram is shifted towards the low conductance values (for $\gamma < 0$) when decreasing k_{0f}. Similarly, a molecular junction with a smaller k_{0r} will get longer elongated before rupture, allowing the sampling of electrode gaps with an associated lower conductance (for $\gamma < 0$) (Fig. S2b).

The electrode gap at mechanical equilibrium (ξ_{eq}) indicates how long a junction needs to be elongated before pulling forces are exerted. As shown in Fig. S2c, a larger ξ_{eq} results in conductance histograms with more contributions from low conductance points (for $\gamma < 0$). This is because increasing ξ_{eq} decreases the force that is being applied to the junction at a given electrode gap, making the junction to rupture at longer elongations.

Finally, changes in the distance from the electrode gap at mechanical equilibrium and the rupture energy barrier ($\chi^{\ddagger}$) effectively change the force-dependent rupture rate (see Eq. 1). Then, varying this parameter causes equivalent trends in the conductance histogram (Fig. S2d-f) as those observed when varying the spontaneous rupture rate (Fig. S2a and b).

III. RECOVERY AND ROBUSTNESS OF THE MICROSCOPIC PARAMETERS FROM FITTING

Extracting all microscopic parameters that define the coefficients c_1-c_4 (Eqs. 9-12) can be achieved by complementing the conductance measurements with force-spectroscopy of both the metal-metal and the molecular junction. Since this data is not currently available, we demonstrate the procedure and its robustness with synthetic data.

Synthetic data for the rupture force spectroscopy (of both the metal-metal contact and the molecular junction) and for the conductance histogram was generated as follows. We first sampled the probability density functions of initial $p_0(\xi_0)$ (Eq. 3) and rupture $p_r(\xi_r)$ (Eq. 4) electrode gaps to generate corresponding sets of initial $\{\xi_0\}$ and rupture $\{\xi_r\}$ electrode gaps using the parameters in Table 1. From this data set, the distribution of rupture forces can be reconstructed by taking into account the elastic constant of the metal-metal or molecular junction (taken to be identical to the one of gold as this is often the softest feature of the junction). The resulting synthetic rupture force histograms are shown in Fig. S3a and b. We then selected random pairs from the ($\{\xi_0\}$, $\{\xi_r\}$) set to generate a set of electrode gap elongation trajectories that was then employed to get a set of sampled conductance values (using Eq. 7) and construct the conductance histograms (Fig. S3c).

To extract the parameters that summarize the mechanical properties of the junction, we fitted the rupture-force histograms using Eq. 2 for both the metal-metal and molecular junction. From this fit, we extracted the rupture rate at zero force (k_0) and the distance to the transition state ($\chi^{\ddagger}$) without using any information about the simulations. In experiments, the inverse temperature β, loading rates ($\dot{F}_f, \dot{F}_r$) and elasticity of the junctions (κ_f, κ_ξ) are known.

To extract the parameters that summarize the conductance properties of the junction, we employed Eq. 8 to fit the synthetic conductance histograms to extract c_1-c_4. To test the robustness of the fitting procedure, we compare the extracted parameters to the original parameters in Table S1 yielding results that are comparable to the original set. Since we now have access to the mechanical parameters, we can now extract the conductance decay coefficient (γ), the molecular base transmission ($\log T_0$), the transmission at mechanical equilibrium ($\log T_{\text{eq}}$) and its corresponding electrode gap (ξ_{eq}) from $c_1 - c_4$. That is, all microscopic parameters can be extracted from two sets of force spectroscopy experiments (one for the metal-metal junction and another one for the molecular junction), and the conductance histogram. Importantly, the extracted parameters exhibit good quantitative agreement with the true original values (see Table 1), showing that the fitting procedure is robust even given that it is highly nonlinear.

In all cases, the fittings were done using the non-linear least squares method, as implemented in the scipy.optimize.curve_fit python package, as exemplified in the script below:

```
import numpy as np
from scipy.optimize import curve_fit

def fit_func_F(F,N,f1,f2,N0):
    "Rupture force probability density function, Eq. 2"
    return N*np.exp(f1*F-f2*np.exp(f1*F)) + N0

```

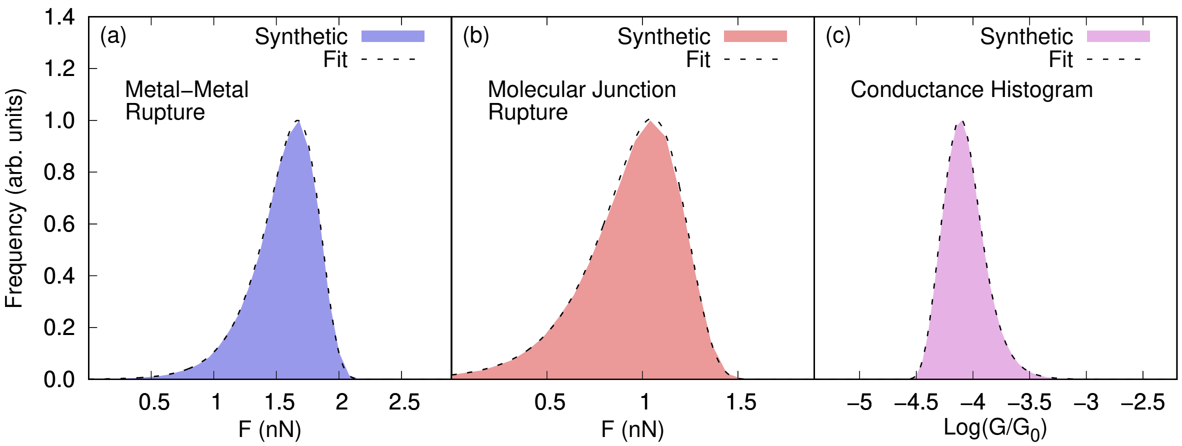

Supplementary Figure S3. Synthetic rupture-force histograms for the (a) metal-metal rupture and (b) molecular junction rupture and their fit to Eq. 2. (c) Numerical conductance histogram and its fit to Eq. 8. The synthetic histograms were generated from 10k pulling trajectories. The parameters resulting from the fitting are shown in tables 1 and S1.

```python
def fit_func_T(g,N,c1,c2,c3,c4,N0):
    "Conductance histogram, Eq. 8"
    return N*(1-np.exp(-c1*(np.exp(c2*g))))*(np.exp(-c3*(np.exp(c4*g))))+ N0

#Guessed parameters for the rupture force histogram fitting
guess_F = [guess_N, guess_f1, guess_f2, guess_N0]

#Rupture force histogram fitting. c_F contain the fitted parameters and
#cov_F the estimated covariance
c_F,cov_F = curve_fit(fit_func_F,forces_file,forces_frequencies_file,guess_F)

#Guessed parameters for the conductance histogram fitting
guess_T = [guess_N, guess_c1, guess_c2, guess_c3, guess_c4, guess_N0]

#Rupture force histogram fitting. c_T contains the fitted parameters and
#cov_T the estimated covariance
c_T,cov_T = curve_fit(fit_func_T,logT_file,logT_frequencies_file,guess_T)
```

Parameter	Original	Fit
c_1	-7.74	-7.76
c_2	2.10×10^{-14}	3.50×10^{-14}
c_3	-7.74	-7.56
c_4	4.20×10^{-13}	1.29×10^{-14}

Supplementary Table S1. Numerical stability of the fit to the conductance histogram. The original values were used to create synthetic conductance histogram that was then fit using Eq. 8. The new extracted parameters are close to the original set and are accurate enough to recover the physical microscopic parameters in Table 1

IV. COMPARISON WITH THE REUTER-RATNER MODEL

In Fig. S4, we contrast fits for the CnSMe series and for the aromatic molecules (A1-N, A2-N and A2-SMe) obtained with the Reuter-Ratner[1, 2] approach with the ones achievable using the theory in this work. The approach by Reuter and Ratner provides expressions for the conductance histograms based on introducing a phenomenological Gaussian distribution of the level alignment and coupling to the electrodes in the Landauer formula for electron transport. The Reuter-Ratner fits were obtained using Eq. 4 from Ref. 2 (equivalent to Eq. 3 in Ref.1) adapted to describe logarithmically binned histograms. The corresponding χ^2 errors and R^2 coefficients are shown in Table S2. In all cases, both approaches yield reasonable fits of the conductance histograms, with the theory presented in this work yielding better fits as measured by χ^2 and R^2. The definite advantage of our strategy is that the origin of conductance dispersion is linked to microscopic features of the free-energy profile of the junction, its mechanical manipulation, and the ability of the molecule to transport charge.

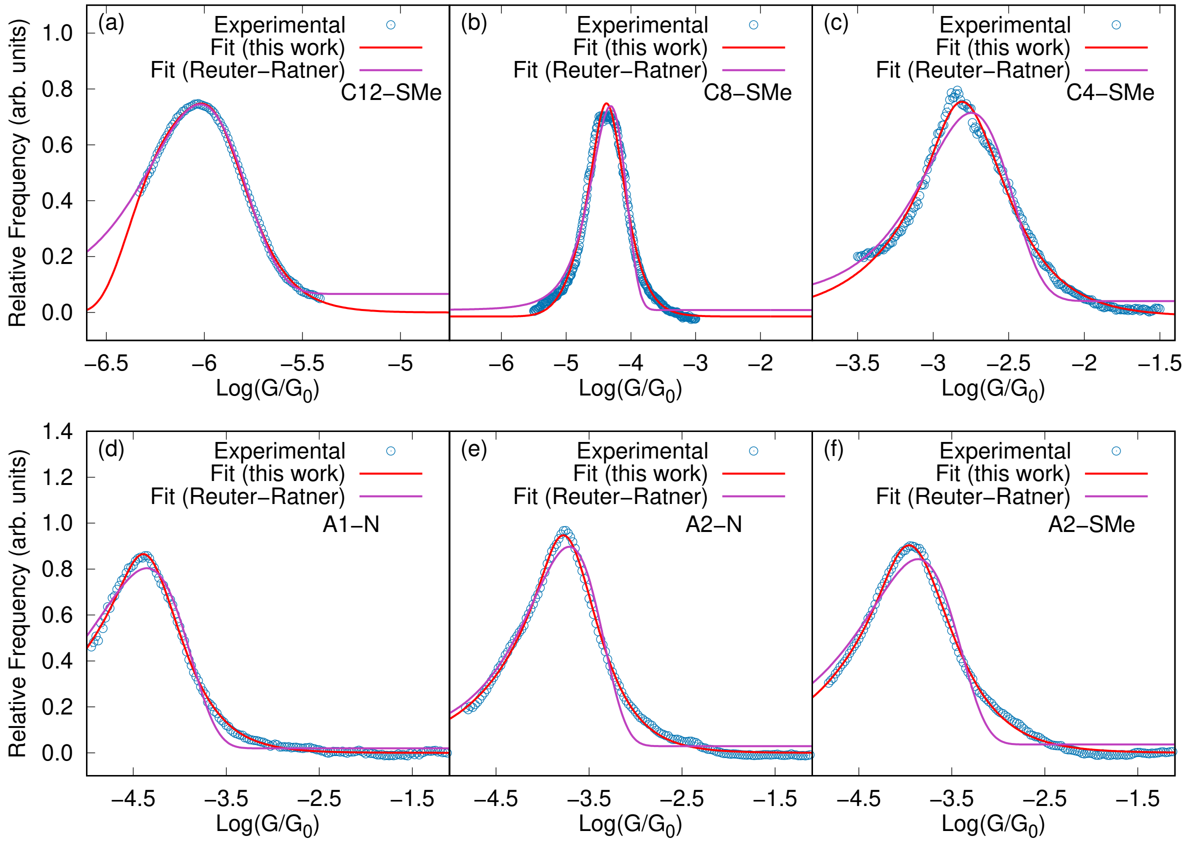

Supplementary Figure S4. Fittings of break-junction conductance histograms in the Cn-SMe series and for the A1-N, A2-N and A2-SMe aromatic molecules using our proposed microscopic theory (Eq. 8) and the phenomenological model by Reuter and Ratner *et al.*[1, 2].

Molecule	R^2 (this work)	χ^2 (this work)	R^2 (Reuter-Ratner)	χ^2 (Reuter-Ratner)
C12-SMe	0.999	6.89×10^{-5}	0.998	1.42×10^{-4}
C8-SMe	0.993	1.38×10^{-3}	0.974	6.12×10^{-3}
C4-SMe	0.993	1.52×10^{-3}	0.966	8.18×10^{-3}
A1-N	0.998	3.98×10^{-4}	0.987	3.72×10^{-3}
A2-N	0.996	1.55×10^{-3}	0.974	2.58×10^{-2}
A2-SMe	0.992	6.30×10^{-4}	0.967	1.26×10^{-2}

Supplementary Table S2. Comparison of the quality of fits for the conductance histograms in the Cn-SMe series and for the A1-N, A2-N and A2-SMe aromatic molecules using the proposed theory and the Reuter-Ratner approach[1, 2] as measured by χ^2 errors and R^2 coefficients.

V. FIT TO MCBJ EXPERIMENTS

Figure S5 shows the fit obtained using Eq. 8 to MCBJ experiments for C8-DT alkanedithiol and the C8-N and C6-N alkanediamines reported in Ref.[3]. In these experiments, different contributions to the conductance histogram were isolated using an unsupervised learning algorithm, resulting in multiple conductance histograms that can be individually fitted to Eq. 8. The resulting fitting parameters are shown in Table S3.

Supplementary Table S3. Parameters describing the experimental conductance histograms in Fig. S5 obtained by fitting to Eq. 8, and R^2 quality of the fit.

Molecule (cluster)	c_1	c_2	c_3	c_4	R^2
C8-DT (1)	-4.64	1.00×10^{-9}	-5.98	1.507×10^{-13}	0.996
C8-DT (2)	-4.15	8.13×10^{-10}	-3.04	8.52×10^{-9}	0.970
C8-DT (3)	-5.62	3.17×10^{-11}	-7.47	2.15×10^{-17}	0.999
C8-DT (4)	-1.35×10^{1}	6.94×10^{-19}	-0.85	6.86×10^{-1}	0.999
C8-DT (5)	-1.70×10^{1}	1.68×10^{-28}	-1.83	1.71×10^{-2}	0.992
C8-DT (6)	-1.82×10^{1}	6.28×10^{-34}	-1.25	1.09×10^{-1}	0.986
C8-N (1)	-7.66	2.37×10^{-16}	-4.54×10^{-3}	6.94×10^{2}	0.997
C6-N (1)	-6.66	3.64×10^{-12}	-6.52×10^{-3}	3.58×10^{2}	0.995

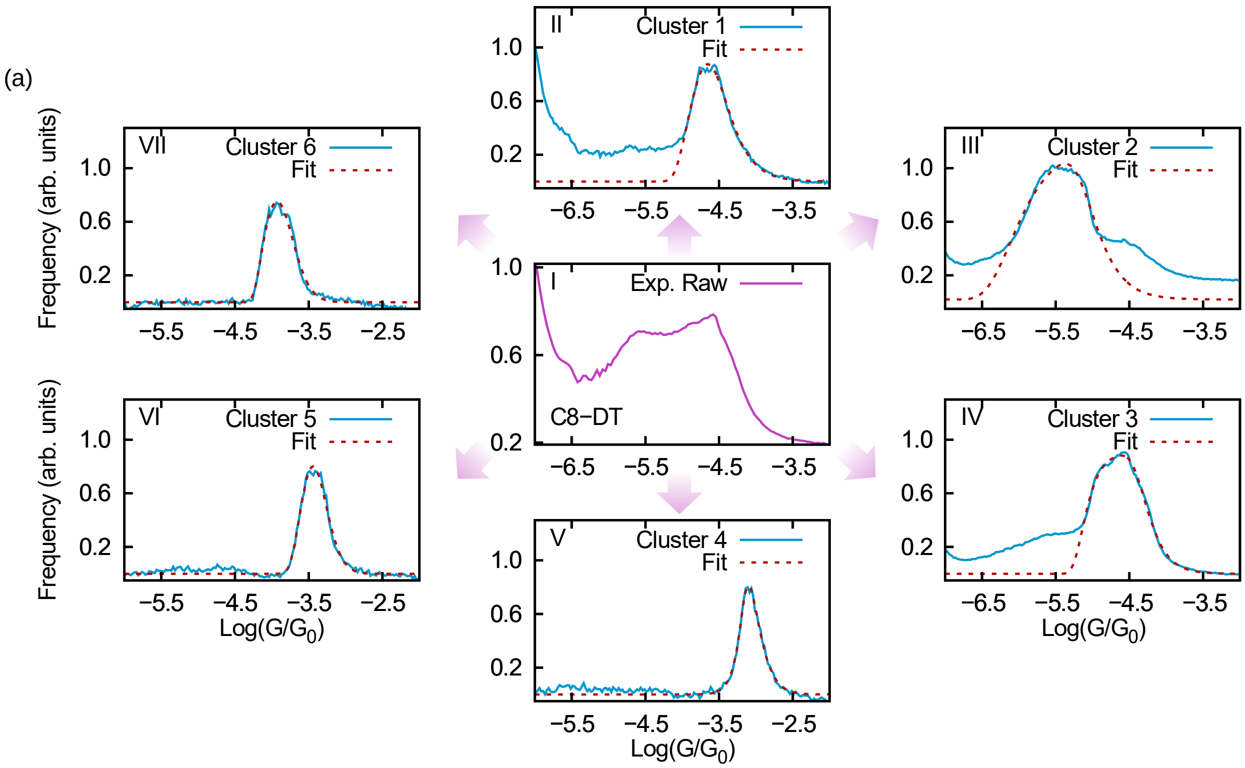

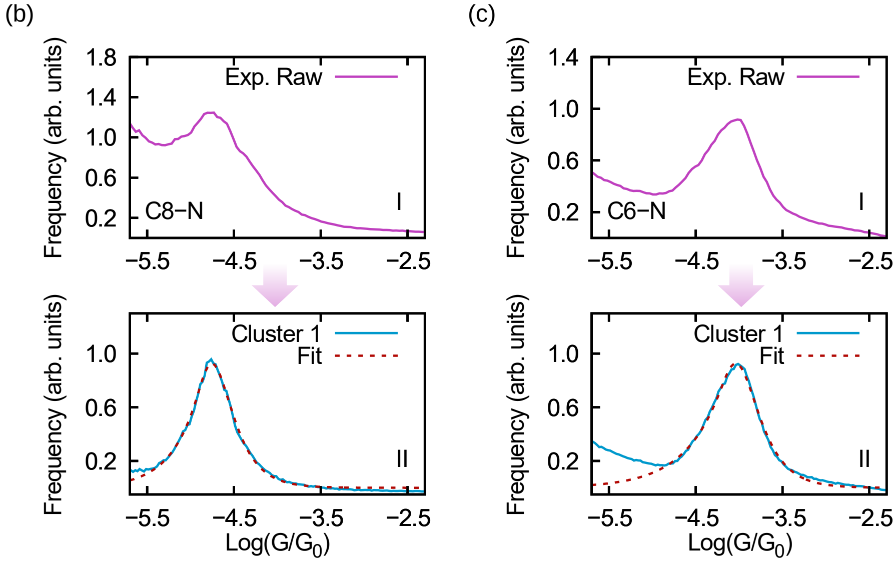

Supplementary Figure S5. MCBJ experimental conductance histograms of junctions containing (a) the C8-DT alkanedithiol and the (b)-(c) C8-N and C6-N alkanediamines and their fit to Eq. 8. In green is the usual experimental conductance histogram, for which clusters of contributions were isolated using an unsupervised learning algorithm. In all cases, the experimental data was obtained from Ref. [3]. The excellent fits indicate that the functional form of Eq. 8 is also applicable to MCBJ.

SUPPLEMENTARY REFERENCES

[1] R. Quan, C. S. Pitler, M. A. Ratner, and M. G. Reuter, Quantitative interpretations of break junction conductance histograms in molecular electron transport, ACS nano **9**, 7704 (2015).

[2] P. D. Williams and M. G. Reuter, Level alignments and coupling strengths in conductance histograms: the information content of a single channel peak, J. Phys. Chem. C **117**, 5937 (2013).

[3] F. H. Van Veen, L. Ornago, H. S. Van Der Zant, and M. El Abbassi, Benchmark study of alkane molecular chains, J. Phys. Chem. C **126**, 8801 (2022).
